# Long-term nitric oxide exposure induces cough hypersensitivity via non-inflammatory activation of the HIF1α–TRPV1 pathway

**DOI:** 10.3389/fphar.2026.1679727

**Published:** 2026-01-19

**Authors:** Jingxin Zhao, Jinjun Jiang, Peifang Zhang, Yue Xiong, Rong Yan, Chuling Zhang, Xuan Zeng, Wenbin Deng, Yichu Nie

**Affiliations:** 1 Clinical Research Center, the First People’s Hospital of Foshan (The Affiliated Foshan Hospital of Southern University of Science and Technology), School of Medicine, Southern University of Science and Technology, Foshan, Guangdong, China; 2 State Key Laboratory of Respiratory Disease, National Clinical Research Center for Respiratory Disease, Guangzhou Institute of Respiratory Health, the First Affiliated Hospital of Guangzhou Medical University, Guangzhou, Guangdong, China; 3 Department of Respiratory and Critical Care Medicine, the First People’s Hospital of Foshan (The Affiliated Foshan Hospital of Southern University of Science and Technology), School of Medicine, Southern University of Science and Technology, Foshan, Guangdong, China; 4 The Eighth Affiliated Hospital, Sun Yat-sen University, Shenzhen, Guangdong, China; 5 School of Pharmaceutical Sciences (Shenzhen), Shenzhen Campus of Sun Yat-sen University, Shenzhen, Guangdong, China; 6 State Key Laboratory of Neurology and Oncology Drug Development, Nanjing, Jiangsu, China

**Keywords:** cough hypersensitivity, HIF1α–TRPV1 signaling, neural sensitization, nitric oxide (NO), noninflammatory cough model

## Abstract

**Background:**

Chronic cough hypersensitivity is common across respiratory diseases and often occurs without airway inflammation, yet effective treatments remain limited. Nitric oxide (NO), an important endogenous signaling molecule and environmental pollutant, has been implicated in respiratory pathophysiology, but its role in cough hypersensitivity remains unclear.

**Aim:**

The aim of this study was to investigate whether long-term NO exposure induces cough hypersensitivity and to define the underlying mechanisms involved.

**Methods:**

A guinea pig model of chronic NO exposure was established and compared with a cigarette smoke (CS) –induced cough model. Cough sensitivity was assessed using capsaicin challenge tests. Airway pathology and inflammation were evaluated by histological staining and molecular analyses *in vivo* and in 16HBE epithelial cells. Expression of TRPV1 and HIF1α was examined in tracheal tissues and ND7/23 sensory neuron-like cells using immunofluorescence and qPCR.

**Results:**

Acute NO exposure did not trigger coughing. Notably, prolonged NO exposure significantly increased capsaicin-induced cough frequency and reduced cough latency. In contrast to CS, chronic NO exposure did not induce airway inflammation, epithelial remodeling, or cytokine upregulation. Instead, NO exposure markedly enhanced the expression of TRPV1 and HIF1α in airway sensory fibers and ND7/23 cells.

**Conclusion:**

These findings demonstrate that prolonged NO exposure induces cough hypersensitivity via HIF1α–TRPV1–mediated neural sensitization, independent of airway inflammation. This study establishes a novel non-inflammatory model of chronic cough and identifies potential therapeutic targets for refractory cough.

## 1 Introduction

Cough is an essential protective reflex that clears inhaled irritants, mucus, and harmful gases from the respiratory tract (Lu H et al., 2024). However, chronic cough, defined as a cough lasting longer than 8 weeks, is one of the most common complaint for outpatient visits in respiratory medicine (Birring SS et al., 2025). Its global prevalence is estimated at 9.6% (Puzzolo E et al., 2024). Although chronic cough is often managed by treating its identifiable causes, the underlying etiology remains unclear in many patients who exhibit cough hypersensitivity (Krüger K et al., 2022). These patients typically experience persistent dry cough induced by multiple irritants, despite normal chest radiographs and the absence of overt airway disease (Morice A et al., 2021). Refractory chronic cough accounts for approximately 15% of chronic cough cases and remains challenging to manage due to limited therapeutic options (Wallace DV, 2023). Thus, effective treatment for chronic cough remains a significant unmet clinical need.

Despite ongoing efforts, progress in understanding and treating chronic cough hypersensitivity has been limited. The main bottleneck in this field is the lack of *in vitro* and *in vivo* models that faithfully recapitulate the clinical features of chronic cough (Grace MS et al., 2013; Mai Y et al., 2020). Among available laboratory species, guinea pigs are the most widely used because their cough reflex closely resembles that of humans (Adner M et al., 2020; Sterusky M et al., 2020). Chronic exposure to cigarette smoke, vehicle exhaust, or cold air has been confirmed to induce chronic cough hypersensitivity in guinea pigs (Lee LY et al., 2010). However, these models consistently exhibit airway inflammation and structural remodeling, which does not fully reflect clinical phenotype of chronic cough hypersensitivity. Thus, it is necessary to develop a chronic cough animal model without obvious pulmonary inflammation.

Nitric oxide (NO) is a small, diffusible gas molecule with diverse physiological and pathological functions in the respiratory tract (Lundberg JO and Weitzberg E, 2022). Endogenously synthesized by the enzymatic degradation of L-arginine via three nitric oxide synthase (NOS) isoforms, NO is primarily produced by airway epithelial and immune cells, where it regulates smooth muscle tone, neurotransmission, and host defense (Pacher P et al., 2007; Ricciardolo FL et al., 2004; Yun HY et al., 1997). Elevated NO levels are characteristic of several respiratory disorders, including asthma and chronic obstructive pulmonary disease (COPD) (Moncada S and Higgs A, 1993). Fractional exhaled nitric oxide (FeNO) was also positively correlated with cough hypersensitivity of patients with bronchial eosinophil infiltration (Maniscalco M et al., 2015). Moreover, simvastatin-induced dry cough has been attributed to NO overproduction via bradykinin release and B2 receptor activation (Khalid S et al., 2014). Despite these associations, the role of chronic NO exposure in cough sensitivity has not been systematically explored, and whether NO can enhance the cough reflex remains unclear.

Sensory neural pathways play a central role in regulating the cough reflex (McGovern AE et al., 2018; Taylor-Clark TE, 2015). The transient receptor potential vanilloid 1 (TRPV1) channel, a non-selective cation channel expressed in airway afferent neurons, is a key mediator of cough reflex sensitivity (Bessac BF and Jordt SE, 2008). Activation of TRPV1 triggers calcium influx and the release of neuropeptides such as calcitonin gene-related peptide (CGRP) and substance P, amplifying cough reflexes (Taylor-Clark TE, 2016). Hypoxia-inducible factor 1α (HIF1α), a transcription factor stabilized under oxidative and nitrosative stress, has been shown to regulate TRPV1 expression (Lacher SE et al., 2023). NO can stabilize HIF1α by inhibiting prolyl hydroxylase activity and modulating redox signaling (Yang Y et al., 2021). Moreover, evidence suggests that NO can activate HIF-1α via S-nitrosylation (Wu KK et al., 2024). Despite these mechanistic links, it remains unknown whether prolonged NO exposure affects cough hypersensitivity by activating the HIF1α–TRPV1 axis.

To address these gaps, this study investigated whether prolonged NO exposure enhances cough sensitivity and sought to uncover the underlying mechanisms. By integrating an *in vivo* guinea pig model with *in vitro* cellular assays, we demonstrate that NO-induced cough hypersensitivity is mediated by HIF1α-TRPV1 driven neural sensitization rather than inflammatory injury. These findings offer new insight into the non-inflammatory mechanisms underlying cough hypersensitivity and highlights potential avenues for developing targeted therapeutic strategies.

## 2 Methods

### 2.1 Animals

Male Hartley guinea pigs (250–300 g, 6–8 weeks old, specific pathogen-free) were obtained from the Experimental Animal Center of Southern Medical University (Guangzhou, China). Animals were acclimatized for 1 week before experiments and maintained under controlled conditions (temperature 22 °C ± 2 °C, relative humidity 50% ± 10%, and a 12-h light/dark cycle) with free access to standard chow and water. For terminal procedures, euthanasia was performed by intraperitoneal injection of sodium pentobarbital (200 mg/kg), followed by cervical dislocation to confirm death. The animal experimental procedures were approved by the Institutional Animal Care and Use Committee of Sun Yat-sen University (SYSU-IACUC-2019-B085).

### 2.2 Animal NO exposure protocol

For NO exposure, guinea pigs were placed in a custom-built chamber with a dynamic gas delivery system. NO gas (15 ppm in air; purity ≥99.5%) was delivered continuously for 24 h per day for 4 weeks. Chamber NO concentrations were continuously monitored using a NO concentration detector to ensure stability and safety. Control animals underwent the same procedure but were exposed to filtered air.

### 2.3 Animal CS exposure protocol

Guinea pigs were exposed to whole-body cigarette smoke twice daily for 21 consecutive days in a chamber as previous reported (Nie YC et al., 2012). In brief, whole-body exposure was conducted in a metal chamber (100 × 60 × 60 cm) with a two-tier rack. Mainstream smoke was generated by a combustion/injection unit consisting of 12 cigarette holders, a W-ye manifold, and a 300 mL glass injector, and delivered into the chamber through one-way valves. Up to 12 cigarettes were combusted per ∼3-min cycle, after which a top-mounted ventilation module purged residual smoke and replenished the chamber with filtered air (purge time ≤15 min). Chamber conditions were continuously monitored with inline sensors and maintained at 19 °C–23 °C, O_2_ 19.2%–20.5%. Normal controls were placed in an identical chamber with filtered air.

### 2.4 Cough challenge test

Cough challenge tests were performed using a BUXCO system (Buxco, Data Sciences International, New York, United States) following aerosol stimulation with capsaicin (CAP, TargetMol, Boston, MA, United States) or sodium nitroprusside (SNP, Beyotime, Shanghai, China). Guinea pigs were placed in a transparent plethysmograph chamber connected to an ultrasonic nebulizer, and aerosolized CAP (50 μM in 0.9% saline containing 0.01% polysorbate 80, TargetMol, Boston, MA, United States) or SNP (100 mg/mL in 0.9% saline, Beyotime, Shanghai, China) was administered for 3 min at a flow rate of 1.2 mL/min. Cough events were recorded for 10 min from the onset of nebulization. Cough events were defined as forceful expiratory reflexes characterized by a sharp expiratory airflow peak accompanied by distinct body movement, whereas sneezes appeared as rapid, shallow expirations with minimal body motion. All events were confirmed by two independent observers blinded to treatment. Only events meeting both airflow and behavioral criteria were classified as coughs. Inter-observer agreement exceeded 95%. Cough latency was defined as the time from the start of nebulization to the first confirmed cough event.

### 2.5 Histological staining

After the exposure period, guinea pigs were euthanized as described above. Distal trachea (just proximal to the carina) and lung tissues were collected, fixed in 4% paraformaldehyde (Servicebio, Wuhan, China), embedded in paraffin, and sectioned at 5 μm. Sections were stained with hematoxylin and eosin (HE, Servicebio, Wuhan, China) to assess tissue morphology and with periodic acid–Schiff (PAS, Servicebio, Wuhan, China) to evaluate mucus production. Images were obtained using a Ts2-FL Inverted Routine Microscope (Nikon, Tokyo, Japan).

HE-stained lung sections were used to evaluate airway inflammation. For each animal, five non-overlapping fields were selected for inflammatory cell infiltration scoring. Inflammatory infiltration was assessed using a standardized semi-quantitative scoring system, in which peribronchial inflammatory cell accumulation was graded on a 0–3 scale: 0 = no detectable inflammatory cells; 1 = mild, scattered inflammatory cells; 2 = moderate, focal or cuff-like infiltrates; 3 = severe, diffuse or dense cuff-like infiltrates. All sections were scored independently by two blinded observers, and the mean score for each animal was used for statistical analysis. Quantitative data are presented as mean ± SEM, with each animal considered as one biological replicate. Group comparisons were performed using one-way ANOVA followed by Tukey’s *post hoc* test. A p-value <0.05 was considered statistically significant.

Epithelial thickness was quantified on H&E-stained tracheal sections. For each group, three guinea pigs were analyzed. In each animal, 15–25 non-overlapping regions of interest (ROIs) were randomly selected along the ventro-lateral wall. Within each ROI, epithelial thickness was measured in ImageJ (v1.54 g, NIH, Bethesda, MD, United States) as the perpendicular distance from the basement membrane to the epithelial surface, excluding cilia and intraluminal mucus. The mean thickness from all ROIs within an animal was used as the biological replicate for statistical analysis. Measurements were performed by a blinded observer.

PAS staining was quantified in tracheal sections from 3–4 guinea pigs per group as the PAS-positive area fraction (%) within the epithelium using ImageJ. Images were analyzed blinded; ROIs were restricted to the epithelial layer, with intraluminal mucus excluded. After background subtraction, a single global threshold was applied uniformly across all groups, and the mean PAS-positive fraction from all ROIs within each animal was used as the biological replicate for statistical analysis.

### 2.6 Cell culture

The human bronchial epithelial cell line 16HBE and the neuroblastoma × dorsal root ganglion fusion cell line ND7/23 were maintained in DMEM (Gibco, Carlsbad, CA, United States) supplemented with 10% fetal bovine serum (FBS, Gibco, Carlsbad, CA, United States) and 1% penicillin–streptomycin (Gibco, Carlsbad, CA, United States of America) at 37 °C in a humidified atmosphere containing 5% CO_2_. For NO donor exposure, cells were treated with Spermine NONOate (10 μM, MCE, Shanghai, China), freshly prepared in culture medium and incubated for 24 h. The commercial filtered cigarettes (12 mg of tar and 1.2 mg of nicotine per cigarette) were used for cigarette smoke extract (CSE) collection by a modified method as previously published (Hoshino Y et al., 2001). Briefly, CSE was prepared by bubbling the smoke from one commercial cigarette (Baisha, China Tobacco Hunan Industrial CO., LTD., Hunan, China) into 10 mL of serum-free medium, filtered (0.22 μm), and used within 30 min at a final concentration of 5% v/v. Control cells received vehicle treatment only.

### 2.7 Immunofluorescence staining

Paraffin-embedded sections and fixed cells were permeabilized with 0.1% Triton X-100 (Sigma-Aldrich, St. Louis, MO, United States), blocked with 3% bovine serum albumin (BSA), and incubated overnight at 4 °C with primary antibodies against TRPV1, HIF1α, iNOS, NF-κB (antibody details in Supplementary Material). After washing, samples were incubated with species-appropriate fluorescent secondary antibodies and counterstained with DAPI. Fluorescent images were acquired using a Biotek Cytation5 Cell Imaging Multimode Reader (Agilent, Waldbronn, Germany), a Ts2-FL Inverted Routine Microscope, and an LSM480 confocal microscope (Zeiss, Oberkochen, Germany). Positive signals were quantified with ImageJ. Immunofluorescence intensity of TRPV1 and HIF1α on tissue sections was quantified as follows. TRPV1 and HIF1α immunofluorescence was imaged under fixed acquisition settings across groups. For each group, n = 3 animals were analyzed. For each animal, 10–15 non-overlapping ROIs were systematically sampled along the subepithelial region. A binary mask was generated from the PGP9.5 channel in ImageJ using a fixed threshold, and the mask was applied to the TRPV1 channel using the Image Calculator function, yielding an image containing only TRPV1 signal within PGP9.5-positive nerve terminals. The mean gray value within the masked regions was recorded. For immunofluorescence intensity analysis in 16HBE and ND 7/23 cells, images for Control, CSE, and NOE were acquired under identical imaging conditions. Raw files were used for analysis; display LUT/contrast adjustments were applied only to presentation copies and not to analysis images. Images were processed in ImageJ. For each image, single-cell ROIs were manually delineated, and the mean gray value of the target channel was recorded. Per image, ∼40–60 cells were sampled; ≥3 images were analyzed per biological replicate. Three independent experiments (n = 3) were performed. Analyses were conducted blinded to group allocation.

### 2.8 EdU proliferation assay

Cell proliferation was assessed using the EdU (5-ethynyl-2′-deoxyuridine) incorporation assay with a commercial EdU Cell Proliferation Kit (Beyotime, Shanghai, China), following the manufacturer’s instructions. Briefly, cells were incubated with 10 μM EdU for 2 h, fixed with 4% paraformaldehyde, permeabilized, and stained with Click-iT reaction cocktail. Nuclei were counterstained with Hoechst 33,342. The percentage of EdU-positive cells was calculated by quantifying EdU-labeled and total nuclei in five random microscopic fields per group using a Ts2-FL Inverted Routine Microscope.

### 2.9 Quantitative real-time PCR (qPCR)

Total RNA was extracted using an RNA-Quick Purification Kit (ES Science, Shanghai, China), andRNA concentration and purity were assessed using a NanoDrop spectrophotometer (Thermo Fisher, Waltham, MS, United States). cDNA was synthesized from 1 μg of total RNA using a reverse transcription kit (TransGen Biotech, Beijing, China) following the standard protocol. Quantitative PCR was performed with qPCR SuperMix (TransGen Biotech, Beijing, China), with primers sequences listed in Supplementary Table S2. The relative mRNA expression levels were calculated and normalized to GAPDH. Each sample was run in triplicate.

### 2.10 Calcium imaging

Intracellular Ca^2+^ levels were measured using the fluorescent calcium indicator Fluo-4 a.m. (Beyotime, Shanghai, China). ND7/23 cells were seeded onto glass-bottom dishes and treated with Spermine NONOate, CSE or vehicle for 24 h. Following treatment, cells were incubated with Fluo-4 a.m. for 30 min at 37 °C in the dark. Fluorescence images were acquired using a Ts2-FL Inverted Routine Microscope under fixed exposure settings across all groups. For each biological replicate, 3–5 non-overlapping fields were imaged. Image analysis was performed using ImageJ. Mean fluorescence intensity was quantified from manually delineated single-cell ROIs (∼40–60 cells per image). The per-replicate mean fluorescence intensity was used for statistical comparison.

### 2.11 Statistical analysis

All data are presented as mean ± standard error of the mean (SEM). Statistical analysis was performed using GraphPad Prism 9.0 (GraphPad Software, San Diego, CA, United States). For comparisons between two groups, unpaired Student’s t-tests were used. For multiple group comparisons, one-way ANOVA followed by Tukey’s *post hoc* test was applied. A *P*-value <0.05 was considered statistically significant.

## 3 Results

### 3.1 NO is not a cough trigger, but long-term NO exposure induces cough hypersensitivity in Guinea pigs

**FIGURE 1:**
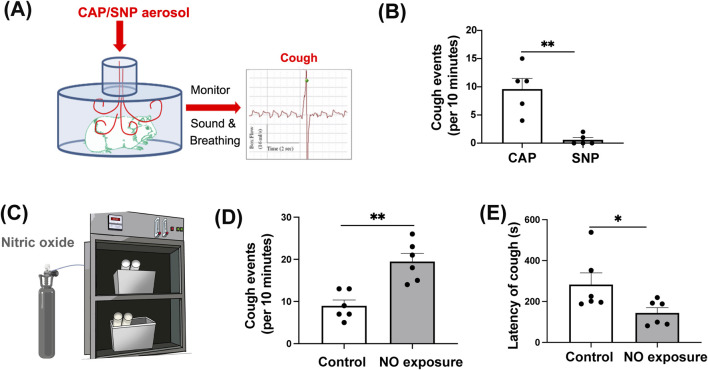
NO stimulation does not induce coughing, and long-term NO exposure increases cough sensitivity in guinea pigs. **(A)** Cough detection setup for guinea pigs after capsaicin and SNP nebulization. **(B)** Cough frequency in guinea pigs after capsaicin and SNP nebulization (*n* = 5; mean ± SEM; **P = 0.0016; two-tailed unpaired Student’s t-test). **(C)** NO exposure chamber setup for guinea pigs. **(D)** Cough frequency in guinea pigs recorded after 4 weeks of NO exposure, following capsaicin stimulation (*n* = 6; mean ± SEM; **P = 0.0012; two-tailed unpaired Student’s t-test). **(E)** Latency to the first cough in guinea pigs after 4 weeks of NO exposure, recorded following capsaicin stimulation (*n* = 6; mean ± SEM; *P = 0.0493; two-tailed unpaired Student’s t-test).

To investigate whether NO directly triggers the cough reflex, we first compared the effects of nebulized sodium nitroprusside (SNP, a NO donor) and capsaicin in guinea pigs. As expected, SNP failed to evoke a significant cough response, whereas capsaicin reliably induced frequent coughing (Figures 1A,B; t (8) = 4.666, *P* = 0.0016), suggesting that NO is not an acute tussive stimulus. To further assess whether chronic NO exposure affects cough sensitivity, we established a NO exposure device (Figure 1C), enabling continuous delivery NO. Following 4 weeks of exposure, capsaicin-evoked cough frequency was significantly increased in NO-exposed groups guinea pigs compared with controls (Figure 1D; t (10) = 4.470, *P* = 0.0012), consistent with the increased coughs sensitization observed in guinea pigs exposed to cigarette smoke (Supplementary Figure S1; t (9) = 2.450, *P* = 0.0368). Additionally, the cough latency was markedly shortened in the NO-exposed guinea pigs (Figure 1E; t (10) = 2.236, *P* = 0.0493). Together, these findings suggest that although direct NO does not induce coughing, prolonged NO exposure markedly increases cough sensitivity in guinea pigs.

### 3.2 Unlike CS exposure, long-term NO exposure does not induce pulmonary inflammation and abnormal repair in the lungs

**FIGURE 2:**
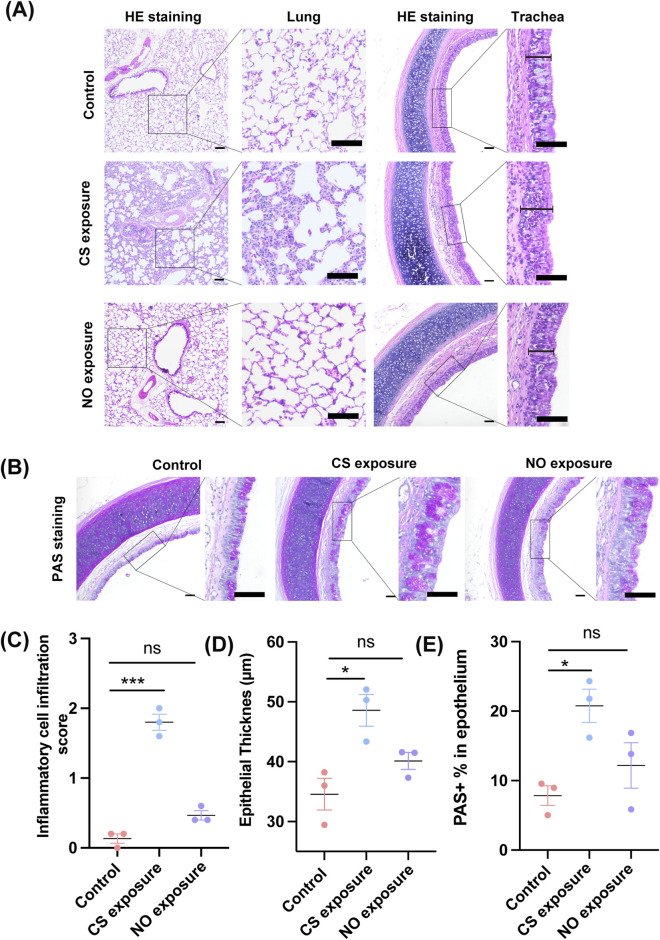
Long-term exposure to NO in guinea pigs induces significant airway and tracheal inflammatory responses, accompanied by abnormal thickening of the airway walls. **(A)** Representative HE staining images of the lungs and trachea from the control group, cigarette smoke-exposed group, and NO exposure group. Scale bar = 50 μm. **(B)** Representative PAS staining images of the lungs and trachea from the control group, cigarette smoke-exposed group, and NO exposure group. Scale bar = 50 μm. **(C)** Semi-quantitative inflammatory cell infiltration scores (*n* = 3; mean ± SEM; one-way ANOVA with Tukey’s *post hoc* test, ***P < 0.0001 vs. control group). **(D)** Statistical analysis of airway epithelial thickness in each group of guinea pigs (*n* = 3; mean ± SEM; one-way ANOVA with Tukey’s *post hoc* test, *P = 0.0122 vs. control group). **(E)** Statistical analysis of PAS-positive area in the airway epithelium of each group of guinea pigs (*n* = 3; mean ± SEM; one-way ANOVA with Tukey’s *post hoc* test, *P = 0.0243 vs. control group).

CS-exposed guinea pig models are widely used in cough research because CS reliably induced airway inflammation and epithelial remodeling, both of which contribute to cough hypersensitivity (Zhong S et al., 2015). To determine whether the enhanced cough sensitivity observed after prolonged NO exposure involves similar inflammatory pathways, we performed histological analyses on lung and tracheal tissues from control, CS-exposed, and NO-exposed guinea pigs. HE staining revealed pronounced epithelial thickening and inflammatory cell infiltration in the CS exposure, consistent with previous established smoke-induced airway injury (Figures 2A,C, *P* < 0.0001). In contrast, tissues from NO-exposed animals exhibited no notable inflammatory changes or structural abnormalities compared with controls (Figures 2A,C, *P* = 0.0754). Airway epithelial thickness was significantly increased in the CS group but unchanged in the NO-exposed group relative to controls (Figure 2C; F (2, 6) = 9.337, *P* = 0.0144; CS vs. Control, *P* = 0.0122; NO vs. Control, *P* = 0.2778). PAS staining further demonstrated marked mucus hypersecretion and goblet cell hyperplasia in the CS group, whereas NO exposure did not alter epithelial mucus content relative to controls (Figure 2B). Quantification showed a significant increase in PAS-positive area in CS-exposed animals but no significant difference between NO-exposed and control groups (Figure 2D; F (2, 6) = 6.985, *P* = 0.0271; CS vs. Control, *P* = 0.0243; NO vs. Control, *P* = 0.4766). Together, these findings indicate that chronic NO exposure does not induce inflammation, epithelial remodelling, or mucus hypersecretion, distinguishing it from the pro-inflammatory effects of cigarette smoke. This suggests that NO-induced cough hypersensitivity arises through a non-inflammatory mechanisms, rather than classical inflammatory injury.

### 3.3 NO exposure does not induce inflammation or abnormal epithelial proliferation in 16HBE cell

**FIGURE 3:**
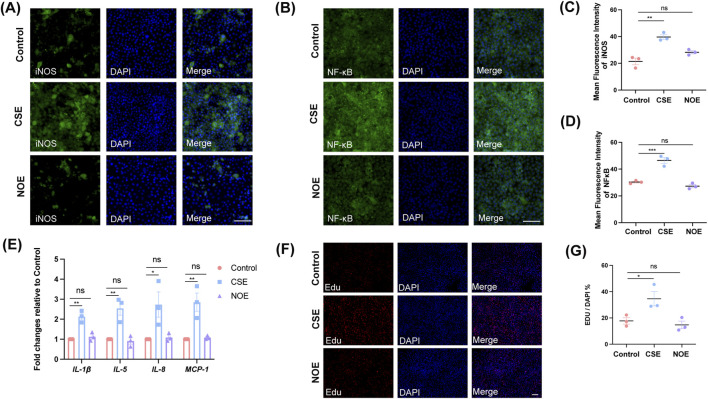
Unlike cigarette smoke exposure, NO incubation does not induce inflammatory responses or abnormal proliferation in 16HBE cells. **(A)** Immunofluorescence staining of iNOS in 16HBE cells from the control group, cigarette smoke extract (CSE) group, and NO exposure (NOE) group. Representative images show the expression and localization of iNOS in each group. Scale bar = 50 μm. **(B)** Immunofluorescence staining of NF-κB in 16HBE cells from the control group, CSE group, and NOE group. Scale bar = 50 μm. **(C)** Quantification of mean fluorescence intensity of iNOS expression from each group (*n* = 3; mean ± SEM; one-way ANOVA with Tukey’s *post hoc* test, **P = 0.0012 vs. control group). **(D)** Quantification of mean fluorescence intensity of NF-κB expression from each group (*n* = 3; mean ± SEM; one-way ANOVA with Tukey’s *post hoc* test, ***P = 0.0002 vs. control group). **(E)** Relative mRNA expression levels of IL-1β, IL-5, IL-8, and MCP-1 were measured in 16HBE cells treated with CSE or NO for 48 h. Gene expression levels were normalized to GAPDH and are presented as fold change relative to the control group (*n* = 3; mean ± SEM; one-way ANOVA with Tukey’s *post hoc* test; **P = 0.0014 for IL-1β, **P = 0.0062 for IL-5, *P = 0.0384 for IL-8, **P = 0.0064 for MCP-1 vs. control group). **(F)** Representative images of EdU staining in 16HBE cells from the control group, cigarette smoke exposure group, and NO exposure group. Scale bar = 50 μm. **(G)** Quantification of the ratio of EdU-positive cells to total cells in 16HBE cells from each group (*n* = 3; mean ± SEM; one-way ANOVA with Tukey’s *post hoc* test, *P = 0.0476 vs. control group).

To further verify the absence of inflammatory responses observed *in vivo*, we examined the effects of NO exposure on airway epithelial cells *in vitro*. 16HBE cells were treated with either NO donor or cigarette smoke extract (CSE) for 24 h, and inflammatory markers was assessed. Immunofluorescence staining revealed that CSE significantly elevated the expression levels of inducible nitric oxide synthase (iNOS) and nuclear factor-kappa B (NF-κB), indicating a strong inflammatory response (Figures 3A,B). In contrast, NO-treated cells exhibited no significant increase in iNOS or NF-κB expression compared to control (Figures 3A,B). Quantitative analysis confirmed significant increases in fluorescence intensity of iNOS and NF-κB in the CSE group (Figures 3C,D; iNOS: F (2, 6) = 23.61, *P* = 0.0014; CS vs. Control, *P* = 0.0012; NF-κB: F (2, 6) = 46.12, *P* = 0.0002; CS vs. Control, *P* = 0.0007), whereas NO exposure produced no significant change (Figures 3C,D, *P* = 0.0962 for iNOS and *P* = 0.3929 for NF-κB). To extend these observations to the transcriptional level, we performed qPCR analysis of pro-inflammatory cytokines IL-1β, IL-5, IL-8, and MCP-1 after 48 h of treatment. Consistent with previous results, CSE significantly upregulated all four cytokines (Figure 3E; IL-1β: F (2, 6) = 26.50, *P* = 0.0011; CS vs. Control, *P* = 0.0014; IL-5: F (2, 6) = 17.23, *P* = 0.0033; CS vs. Control, *P* = 0.0062; IL-8: F (2, 6) = 6.825, *P* = 0.0285; CS vs. Control, *P* = 0.0384; MCP-1: F (2, 6) = 15.42, *P* = 0.0043; CS vs. Control, *P* = 0.0064), whereas NO exposure did not alter cytokine expression (Figure 3E, IL-1β: *P* = 0.7495; IL-5: *P* = 0.9598; IL-8: *P* = 0.9832; MCP-1: *P* = 0.9732). We next investigated whether NO exposure affects epithelial proliferation responses. EdU assays revealed a significant increase in proliferative activity in CS-treated cells (Figures 3F,G; F (2, 6) = 7.819, *P* = 0.0213; CS vs. Control, *P* = 0.0476). In contrast, NO exposure did not alter the proportion of EdU-positive cells compared with controls (Figures 3F,G, *P* = 0.8434), suggesting that NO does not stimulate aberrant epithelial proliferation. Together, these *in vitro* findings mirror our *in vivo* observations and demonstrate that NO exposure, unlike cigarette smoke, does not induce airway inflammation, cytokine induction, or epithelial remodelling. These results reinforce the conclusion that NO-induced cough hypersensitivity arises through a non-inflammatory mechanism, distinct from classical smoke-induced inflammatory pathways.

### 3.4 Consistent with CS exposure-induced cough hypersensitivity, NO exposure induces increased expression of TRPV1 and HIF1α

**FIGURE 4:**
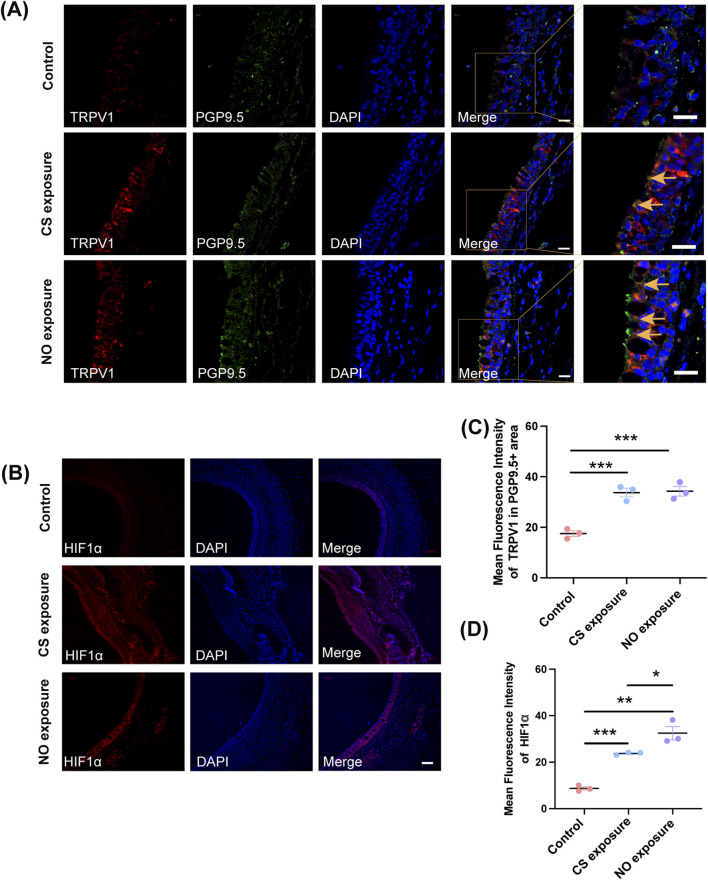
Long-term NO exposure increases TRPV1 and HIF1α expression in the airways of guinea pigs. **(A)** Immunofluorescence images showing TRPV1 and PGP9.5 expression in the airways of the control group, cigarette smoke-exposed group, and NO-exposed group. Arrows indicate PGP9.5-positive nerve terminals. Scale bar = 20 μm. **(B)** Immunofluorescence images showing HIF1α expression in the airways of the control group, cigarette smoke-exposed group, and NO-exposed group. Scale bar = 100 μm. **(C)** Quantification of mean fluorescence intensity of TRPV1 expression in PGP9.5 positive area of each group (*n* = 3; mean ± SEM; one-way ANOVA with Tukey’s *post hoc* test, ***P = 0.0010 for CS exposure group and ***P = 0.0008 for NO exposure group vs. Control group). **(D)** Quantification of mean fluorescence intensity of HIF1α expression in the airways of each group (*n* = 3; mean ± SEM; one-way ANOVA with Tukey’s *post hoc* test, ***P = 0.0020 for CS exposure group and ***P = 0.0002 for NO exposure group vs. control group).

Cough hypersensitivity is influenced not only by airway inflammation but also by central sensitization and activation of the transient receptor potential (TRP) channels (Rouadi PW et al., 2021). Having established that long-term NO exposure enhances cough sensitivity without eliciting classical inflammatory responses, we next investigated whether this heightened sensitivity involves altered sensory transduction within the airway. Specifically, we focused on TRPV1, a key sensory ion channel implicated in cough reflex modulation, and HIF1α, a transcription factor known to regulate TRPV1 under hypoxic or redox conditions (Kanngiesser M et al., 2014; Soma S et al., 2025). Immunofluorescence staining revealed that TRPV1 expression was markedly increased in the tracheal tissues of guinea pigs exposed to either CS or NO, relative to the control group (Figure 4A). TRPV1 displayed partial colocalization with PGP9.5-positive subepithelial nerve fibers, although additional epithelial signal was also observed. To more specifically assess neuronal TRPV1, quantification restricted to PGP9.5 positive areas showed significantly elevated TRPV1 in both exposure groups (Figure 4C; F (2, 6) = 34.68, *P* = 0.0005; CS vs. Control, *P* = 0.0010; NO vs. Control, *P* = 0.0008). Similarly, HIF1α expression was significantly upregulated in both CS- and NO-exposed animals (Figures 4B,D, F (2, 6) = 48.86, *P* = 0.0002; CS vs. Control, *P* = 0.0020; NO vs. Control, *P* = 0.0002), indicating potential transcriptional activation in response to chronic exposure. These results suggest that although NO exposure does not provoke inflammation or epithelial remodeling, it may enhance cough sensitivity via upregulation of TRPV1 and HIF1α. This molecular profile parallels that observed in the CS-induced cough hypersensitivity but occurs independently of inflammatory signaling, supporting TRPV1 and HIF1α as key mediators linking long-term NO exposure to neural sensitization.

### 3.5 NO directly activates theHIF1α–TRPV1 signalling axis in ND7/23 cells

**FIGURE 5:**
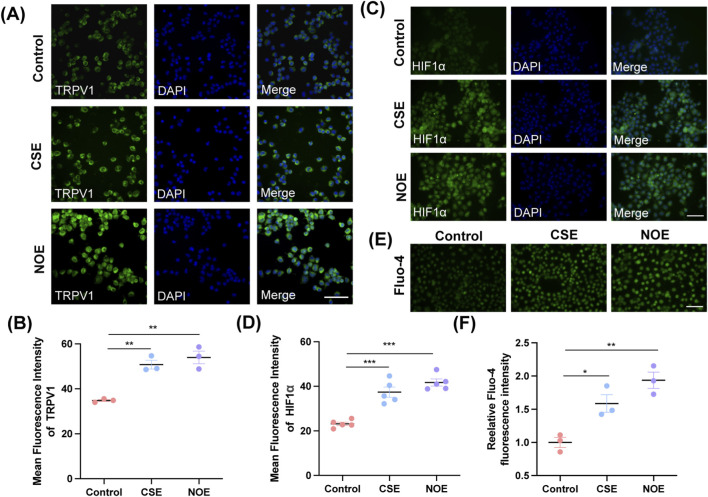
NO exposure increases expression of neural sensitivity–related markers in ND7/23 cells. **(A)** Representative immunofluorescence images of TRPV1 in ND7/23 cells treated with vehicle (Control), cigarette smoke extract (CSE), or Spermine NONOate (NO donor, NOE). Scale bar = 100 μm. **(B)** Quantification of mean fluorescence intensity of TRPV1 expression from each group (*n* = 3; mean ± SEM; one-way ANOVA with Tukey’s *post hoc* test, **P = 0.0033 for CS exposure group and **P = 0.0013 for NO exposure group). **(C)** Representative immunofluorescence images of HIF1α in ND7/23 cells treated with vehicle, CSE, or NOE. Scale bar = 100 μm. **(D)** Quantification of mean fluorescence intensity of HIF1α expression from each group (*n* = 5; mean ± SEM; one-way ANOVA with Tukey’s *post hoc* test, ***P = 0.0001 for CS exposure group and ***P < 0.0001 for NO exposure group vs. control group). **(E)** Representative Fluo-4 a.m. images of ND7/23 cells treated with vehicle, CSE or NOE. Scale bar = 100 μm. **(F)** Quantification of mean fluorescence intensity of Fluo-4 a.m. relative to Control group from each group (*n* = 3; mean ± SEM; one-way ANOVA with Tukey’s *post hoc* test, *P = 0.0246 for CS exposure group and **P = 0.0027 for NO exposure group vs. control group).

To examine whether NO-induced TRPV1 and HIF1α upregulation is neuron-autonomous, ND7/23 sensory neuron-like cells were used. Cells were treated with Spermine NONOate, a nitric oxide donor (Ramamurthi A and Lewis RS, 1997), for 48 h to mimic long-term NO exposure. Immunofluorescence analysis revealed marked increases in both TRPV1 and HIF1α expression in NO-treated cells relative to controls, consistent with the *in vivo* findings. CSE also induced similar upregulation (Figures 5A–D; TRPV1: F (2, 6) = 25.91, *P* = 0.0011; CS vs. Control, *P* = 0.0033; NO vs. Control, *P* = 0.0013; HIF1α: F (2, 12) = 34.45, *P* < 0.0001; CS vs. Control, *P* = 0.0001; NO vs. Control, *P* < 0.0001). To further validate that NO exposure enhances neuronal excitability, we conducted Fluo-4 based calcium imaging in ND7/23 cells. Both NO donor and CSE treatment significantly increased baseline intracellular Ca^2+^ fluorescence relative to controls (Figure 5E). Quantification revealed elevated mean Fluo-4 intensity in both groups (Figure 5F; F (2,6) = 17.39, *P* = 0.0032; CS vs. Control, *P* = 0.0246; NO vs. Control, *P* = 0.0027). These results demonstrate that NO directly increases intracellular Ca^2+^ signaling, supporting a role for NO-mediated neural sensitization independent of inflammatory activation. These findings demonstrate that NO can directly induce TRPV1 and HIF1α upregulation in sensory neuron-like cells, independent of epithelial or immune contributions. Together with the *in vivo* data, this supports the concept that NO enhances cough hypersensitivity through non-inflammatory neural sensitization pathways rather than through classical inflammatory mechanisms.

## 4 Discussion

**FIGURE 6:**
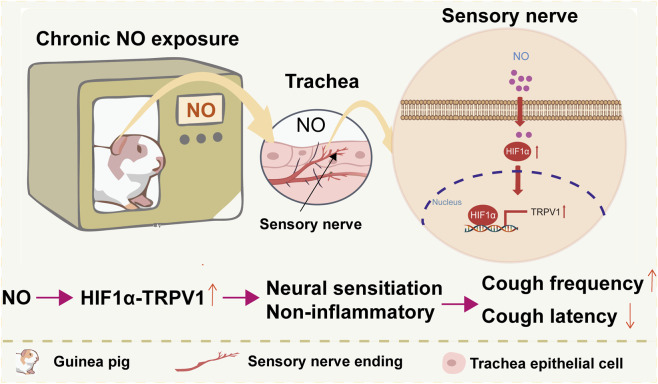
Schematic summary of the proposed mechanism. Long-term NO exposure increases cough sensitivity through HIF1α–TRPV1–dependent neuronal sensitization in tracheal sensory nerve endings, independent of inflammation.

In this study, we investigated the role of NO in regulating cough sensitivity and uncovered a novel non-inflammatory mechanism by which long-term NO exposure enhances cough responsiveness (Figure 6). Although NO is widely recognized as a multifunctional signaling molecule in both physiological and pathological conditions, its direct contribution to cough pathogenesis has remained unclear. Here, we demonstrate that acute stimulation with a NO donor does not trigger coughing in guinea pigs, whereas prolonged NO exposure significantly increases capsaicin-evoked cough frequency and reduces cough latency. Importantly, this enhanced cough sensitivity occurred in the absence of airway inflammation, epithelial remodeling, or cytokines upregulation—features that are typically prominent in cigarette smoke induced models of cough hypersensitivity. Instead, we identified the upregulation of TRPV1 and HIF1αwithin airway sensory structures and in and ND7/23 cells, indicating a pattern of NO-induced neural sensitization. These findings suggest that chronic NO enhances cough sensitivity primarily through modulation of sensory neuronal excitability rather than through inflammatory pathways. These results suggest a possible association between chronic NO exposure and neural sensitization mediated cough hypersensitivity, warranting further investigation into non–inflammatory mechanisms of chronic cough.

In the present study, we established a new cough hypersensitivity guinea pig model by long-term NO exposure. The concentration of NO used in present study is 15 ppm, which is about 200-fold of the FeNO concentration in chronic cough patients (Yi F et al., 2016). We choose this higher concentration of NO for two reasons. On the one hand, to lower NO concentration cannot be precisely and quantitatively controlled on the instrument. And on the other hand, increasing the concentration of NO is a good way to shorten the modelling time. It’s interesting to find that the cough sensitivity induced by NO exposure is gradually decreased during the second to third weeks. This phenomenon also occurred in our previous cough hypersensitivity model of guinea pigs induced by cigarette smoke exposure (Luo YL et al., 2013). We speculate that it is due to the opposite biological effects between short-term NO exposure and long-term NO exposure. And during this period, the physiological effects induced by short-term NO exposure are gradually weakening, while the pathological changes induced by long-term NO exposure are getting stronger day by day. We will further explore the key factor in long-term NO exposure induced cough hypersensitivity and improve our modelling protocol.

Although cigarette smoke exposure models are widely used to investigate cough hypersensitivity, they largely rely on airway inflammation and epithelial remodeling as the major pathological drivers (Li D et al., 2022). Consistent with previous studies, our findings confirmed that CS exposure induced pronounced airway epithelial thickening, mucus hypersecretion, and upregulation of inflammatory markers including iNOS, NF-κB, and the pro-inflammatory cytokines IL-1β, IL-5, IL-8, MCP-1. These changes collectively reflect classical inflammatory responses that induce cough hypersensitivity through tissue injury and immune-mediated sensitization. In contrast, chronic NO exposure did not elicit histological evidence of airway inflammation or epithelial remodeling in guinea pigs, nor did it elicit inflammatory signaling or aberrant proliferation in 16HBE cells. Notably, NO failed to upregulate iNOS, NF-κB, or cytokine expression, clearly distinguishing its effects from those of CS. Instead, NO exposure was associated with the upregulation of TRPV1 and HIF1α in both airway tissues and sensory neuron–like cells, supporting a non–inflammatory mechanism of action. These findings suggest that NO enhances cough sensitivity through modulation of neural plasticity and peripheral sensory sensitization rather than through canonical inflammatory pathways. This divergence underscores the potential role of NO as a neuromodulator capable of amplifying cough reflex sensitivity via mechanisms distinct from those activated in inflammation-driven models.

The upregulation of TRPV1 and HIF1α in NO-exposed guinea pigs and ND7/23 cells suggests that the HIF1α–TRPV1 signaling axis may serve as a central mediator of NO-induced neural sensitization. Although ND7/23 cells originate from dorsal root ganglion neurons and do not fully capture the molecular diversity of vagal sensory neurons that innervate the airway, they retain key nociceptive signaling components—TRPV1 and HIF1α—making them a suitable reductionist model for probing conserved redox-dependent mechanisms independent of inflammatory confounders. TRPV1 is a well-characterized nociceptive ion channel that modulates cough reflex sensitivity by calcium influx in airway afferents following chemical or thermal stimulation (Harford TJ et al., 2021). Its upregulation following NO exposure indicates enhanced neuronal excitability as a likely contributor to cough hypersensitivity. HIF1α, a transcription factor stabilized under hypoxic or nitrosative stress, has been implicated in the regulation of TRPV1 (Che H et al., 2025). Prior studies have demonstrated that NO stabilizes HIF1α protein by inhibiting prolyl hydroxylases activity and through direct S-nitrosylation, thus extending its half-life and promoting downstream gene transcription (Khan M et al., 2015; Li F et al., 2007; Moncada PS, 2015; Sandau KB et al., 2001; Sun N et al., 2024). In airway sensory pathways, such stabilization may act upstream of TRPV1 in airway sensory pathways, promoting transcriptional programs that enhance neuronal excitability (Taylor-Clark TE, 2016). Our observation that Spermine NONOate induced coordinated increases in HIF1α and TRPV1 in ND7/23 cells supports a neuron-antonomous mechanism by which NO potentiates sensory signaling. Notably, these effects occurred in the absence of inflammatory signaling, indicating that NO acts primarily through redox-dependent pathways rather than cytokine-mediated mechanisms. Thus, although CS- and NO-induced cough hypersensitivity arise from distinct upstream processes—inflammatory/oxidative injury for CS versus redox signaling for NO—both appear to converge on heightened excitability of airway afferent neurons via the HIF1α–TRPV1 axis. While this study focused on TRPV1, other sensory mediators such as CGRP and P2X3also contribute to cough reflex modulation and neurogenic signaling (Giniatullin R and Nistri A, 2023; Ruby HA et al., 2024; Wang J et al., 2022). These pathways may operate with HIF1α–TRPV1 axis under chronic NO exposure and warrant further investigation. Together, our findings identify the HIF1α–TRPV1 axis as a critical driver of NO-induced neural plasticity, offering a mechanistic framework distinct from inflammation centric models of cough hypersensitivity. However, it should be emphasized that these data are correlative and do not yet establish that HIF1α–TRPV1 signaling is necessary or sufficient for NO-induced cough hypersensitivity.

The findings of this study provide new insight into how NO modulates cough sensitivity, revealing a non-inflammatory neural mechanism that differs fundamentally from classical cigarette smoke–driven pathways. Given that NO is both endogenously produced in the airway and abundant in polluted or industrial environments, prolonged low-level exposure may represent an underappreciated contributor to cough hypersensitivity in vulnerable populations. Our findings further suggest that targeting the HIF1α–TRPV1 signaling axis could offer therapeutic benefit, particularly for patients with chronic cough who respond poorly to conventional anti-inflammatory or bronchodilator therapies.

Despite these promising findings, several limitations of our study should be acknowledged. First, although we identified a strong association between NO exposure and the upregulation of TRPV1 and HIF1α, the present data do not yet demonstrate that the HIF1α–TRPV1 axis is necessary for this phenotype. We did not test whether pharmacological blockade of TRPV1 (e.g., capsazepine) or HIF1α (e.g., YC-1, PX-478) can reverse NO-induced cough hypersensitivity *in vivo*, nor did we perform loss-of-function studies such as siRNA/shRNA-mediated HIF1α knockdown in ND7/23 cells to determine whether HIF1α is required for TRPV1 upregulation. These functional approaches, together with electrophysiological recordings of airway sensory neurons, will be essential in future work to establish mechanistic causality and to validate HIF1α as an upstream regulator of TRPV1 in NO-induced neural sensitization. Beyond the HIF1α–TRPV1 axis, it is also plausible that NO influences other neurogenic pathways known to regulate cough reflex sensitivity. Neuropeptides such as CGRP and purinergic P2X3 receptors play central roles in sensory neuron excitability and have been implicated in multiple forms of chronic cough. Although these pathways were not assessed in the present study, our findings do not exclude their involvement. Second, our study employed a single concentration and exposure paradigm. In evaluating whether NO acts as an acute cough inducer, the study employed a single nebulized concentration of SNP. This dose was derived empirically from preliminary range-finding assessments to ensure measurable NO availability while avoiding respiratory suppression. Importantly, guinea pigs exposed to this SNP dose retained intact capsaicin-evoked coughing, suggesting that the concentration did not induce respiratory depression or cyanide-related neurotoxicity. Nonetheless, the use of a single acute SNP concentration represents a limitation, as we cannot exclude potential dose-dependent effects or false-negative outcomes. Future studies incorporating multiple SNP concentrations and direct assessments of toxicity will be necessary to more rigorously determine whether NO possesses acute tussive activity. In our *in vitro* experiments, the NO-donor concentration used for stimulating ND7/23 cells was determined through empirical optimization. The concentration ultimately selected reliably induced measurable TRPV1 and HIF1α upregulation without affecting cell viability. Nevertheless, we acknowledge that using a single optimized dose limits the extrapolation of these findings, and future studies incorporating broader dose–response analyses and alternative NO donors will be needed to fully define physiological and pathological relevance. Recent evidence indicates that epithelial cells can modulate cough reflexes by releasing neuromodulators that activate sensory afferents (Seeholzer LF and Julius D, 2024; Soma S, et al., 2025). Future studies employing cell-type–specific markers or functional inhibition will be necessary to determine whether these epithelial pathways also participate in NO-induced cough hypersensitivity. In addition, although PGP9.5 mask–restricted quantification supports increased TRPV1 expression within neuronal structures, the current imaging resolution does not allow definitive exclusion of epithelial TRPV1 signal. Therefore, while our findings strongly suggest neuronal sensitization, a partial epithelial contribution cannot be ruled out. Higher-resolution imaging modalities and neuron-specific reporter or lineage-tracing approaches will be required to further refine TRPV1 localization *in vivo*. In addition, our conclusion that NO upregulates TRPV1 and HIF1α expression is based primarily on immunofluorescence analysis. Although immunofluorescence provides essential spatial information on protein localization within the airway microenvironment, we acknowledge that its quantitative interpretation can be influenced by staining conditions, antibody penetration efficiency, and thresholding parameters. The absence of complementary biochemical validation represents a methodological limitation of the present study. Future work incorporating Western blotting and quantitative PCR will be necessary to strengthen the mechanistic interpretation and fully validate the regulatory effects of NO on these pathways. ND7/23 cells are widely used as a sensory neuron–like model to study conserved mechanisms of TRPV1 regulation and nociceptive signaling in peripheral neurons. While ND7/23 cells provide a practical and well-established sensory neuron–like model, they do not fully recapitulate the complexity of airway afferent neurons, or the central neural circuits involved in cough reflex. Future studies using primary vagal sensory neurons, targeted genetic manipulation, and single-cell profiling—combined with vagal afferent recordings or patch-clamp electrophysiology—will be required to establish cell-type specificity and directly quantify NO-induced changes in neuronal excitability. Despite these limitations, our findings establish a foundational model for exploring non-inflammatory neural modulation of cough sensitivity and highlight the HIF1α–TRPV1 axis as a promising therapeutic target.

## 5 Conclusion

In summary, this study demonstrates that chronic NO exposure enhances cough sensitivity through HIF1α–TRPV1–mediated neural sensitization, independent of airway inflammation. Unlike cigarette smoke, which drives cough hypersensitivity via inflammatory damage, NO acts primarily as a neuromodulator, increasing the excitability of airway sensory pathways without inducing structural or inflammatory damage. By integrating both *in vivo* and *in vitro* evidence, these findings highlight the need for further investigation into NO-driven sensory plasticity and support the HIF1α–TRPV1 axis as a potential therapeutic target for refractory or idiopathic chronic cough.

## Data Availability

The original contributions presented in the study are included in the article/Supplementary Material, further inquiries can be directed to the corresponding authors.
