# Supplementary material for: Long-term nitric oxide exposure induces cough hypersensitivity via non-inflammatory activation of the HIF1α–TRPV1 pathway
Source: Front Pharmacol. 2026 Jan 19;17:1679727. doi: 10.3389/fphar.2026.1679727 (PMC12862251; doi:10.3389/fphar.2026.1679727)
Supplement: Supplementary file 1 [file Supplementaryfile1.docx]

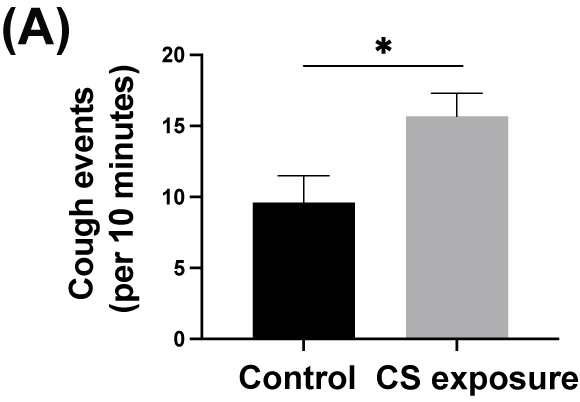


**Figure S1 Cough events per 10 minutes in guinea pigs recorded after 2 weeks of cigarette smoke exposure, following capsaicin stimulation** (*n* = 5-6; mean ± SEM; ** *P* < 0.01; ﻿two-tailed unpaired Student’s t-test).

**Supplementary Table 1** **The antibodies list.**

| **Antibodies** | **Producer** | **Irem No.** | **Dilution** |
| --- | --- | --- | --- |
| Anti-iNOS | Proteintech | 18985-1-AP | 1 : 100 |
| Anti-NF-κB | Abmart | T55034F | 1 : 100 |
| Anti-TRPV1 | NOVUS Biologicals | NBP1-97417 | 1 : 40 |
| Anti-PGP9.5 | Abacm | ab8189 | 1 : 200 |
| Anti- HIF1α | Abcam | ab2185 | 1 : 100 |
| Anti-rabbit IgG (H+L), F(ab')2 Fragment (Alexa Fluor® 488 Conjugate) | CST | 4412 | 1 : 1000 |
| Anti-mouse IgG (H+L), F(ab')2 Fragment (Alexa Fluor® 594 Conjugate) | CST | 8890 | 1:1000 |
| Donkey anti-Rabbit IgG (H+L) Alexa Fluor Plus 594 | Thermo Fisher Scientific | A32754 | 1:1000, |
| Donkey anti-Mouse IgG (H+L) Alexa Fluor 488 | Thermo Fisher Scientific | R37114 | 1: 1000 |

**Supplementary Table 2** The sequence of primers for qPCR.

| **GENE** | **Forward 5’→3’** | **Reverse 5’→3’** |
| --- | --- | --- |
| *GADPH* | TGTTCGTCATGGGTGTGAAC | ATGGCATGGACTGTGGTCAT |
| *IL‑1β* | GCTCCGGGACTCACAGCAAAAA | TTGGGGAACTGGGCAGACTCAA |
| *IL-5* | GTTCCTGGATTACCTGCAAGAA | GTCTCAGCCTTCAATTGTCCAT |
| *IL-8* | CTCCAAACCTTTCCACCCCAA | AAAACTTCTCCACAACCCTCTGC |
| *MCP-1* | GATCTCAGTGCAGAGGCTCG | TTTGCTTGTCCAGGTGGTCC |
